# Examining the Dorsolateral and Ventromedial Prefrontal Cortex Involvement in the Self-Attention Network: A Randomized, Sham-Controlled, Parallel Group, Double-Blind, and Multichannel HD-tDCS Study

**DOI:** 10.3389/fnins.2020.00683

**Published:** 2020-07-14

**Authors:** Víctor Martínez-Pérez, Guillermo Campoy, Lucía B. Palmero, Luis J. Fuentes

**Affiliations:** Department of Basic Psychology and Methodology, University of Murcia, Murcia, Spain

**Keywords:** HD-tDCS, self-prioritization effect, social cognition, shape-label matching task, ventromedial prefrontal cortex, dorsolateral prefrontal cortex

## Abstract

**Background:**

Attention and perception are strongly biased toward information about oneself compared to information about others. The self-attention network, an integrative theoretical framework for understanding the self-prioritization effects (SPE), proposes that the ventromedial prefrontal cortex (VMPFC), and the posterior superior temporal sulcus (pSTS) are the two nodes responsible for the preferential processing of self-related stimuli, which interact with the attentional control network (associated with the dorsolateral prefrontal cortex, DLPFC), responsible for processing other-related stimuli. So far, neuroimaging studies have provided considerable correlational evidence supporting the self-attention network.

**Objective:**

Here we went beyond correlational evidence by manipulating cortical activity using high-definition transcranial direct current stimulation (HD-tDCS), a non-invasive brain stimulation method. We assessed whether anodal and cathodal stimulation of the VMPFC or the DLPFC modulates the processing of self- and other-related stimuli.

**Methods:**

We used an associative unbiased learning procedure, the so-called shape-label matching task, to assess the SPE in a sample of *N* = 90. We accomplished to overcome different methodological weaknesses of previous studies using different multichannel montages for excitatory and inhibitory effects over both the VMPFC and the DLPFC.

**Results:**

We found no effect of shape association for non-matching pairs, whereas there was an effect of shape association in the matching condition. Performance (reaction times and accuracy) was better for the self association than for the other two associations, and performance for the friend association was better than for the stranger association. Thus, we replicated the SPE with behavioral data. At the neural level, none of the stimulation succeeded to modulate the magnitude of the SPE effect.

**Conclusion:**

We discuss the implications of these findings, in particular why cognitive modeling theories about SPEs should favor an epiphenomenal rather than a causal link between VMPFC/DLPFC and the impact of personal significance stimuli on perception.

## Introduction

There is both behavioral and neural evidence about the ubiquitous and pervasive effects of oneself information on attention and perception. In the attentional domain, Moray’s pioneering work in Moray, 1959 on selective attention using Cherry’s shadowing task reported that people automatically direct their attention to an auditory unattended source when their own name is presented there, which was not the case with the others’ names. This bias for one’s own name has been also found with the attentional-blink paradigm (Raymond et al., 1992). As it happens with highly salient stimuli, the attentional blink, i.e., the cost to detect a second target (T2) if a previous one (T1) has been correctly identified, is significantly reduced for one’s name compared to a stranger’s name (Shapiro et al., 1997). Nevertheless, these classic effects of self-priority with the own name have an important methodological limitation because they could be intermingled and confounded with the effects of familiarity, emotional significance, or reward value of the stimuli (Northoff and Hayes, 2011; Sui et al., 2012).

In the perceptual domain, Sui et al. (2012) have developed a new unbiased approach to measure the self-prioritization effect (SPE). In the known as the shape-label matching task, participants have first to learn the association between three geometrical shapes (e.g., triangle, square, and circle) with three different labels (e.g., “you,” “friend,” and “stranger”). In a second step, participants have to judge whether subsequent shape-label pairs were correctly matched or not, according to the previously learned association. The results show an SPE that is reflected in reaction times (RTs) and accuracy, with shorter RTs and better accuracy when the stimuli have been previously associated with self (“you”), compared to those that have been associated with a friend or a stranger. This procedure warrants that the SPE can be attributed neither to an effect of familiarity nor to the concreteness, frequency, or length of the words used (Sui et al., 2012; Humphreys and Sui, 2015).

At the neural level, different neuroimaging studies have shown that self and non-self involve differentiated brain areas in the medial prefrontal cortex (for a review, see Wagner et al., 2012, or the meta-analysis by Denny et al., 2012). Sui et al. (2013) tested the neural networks involved in the SPE. When participants made judgments about the self-tagged stimuli, the ventromedial prefrontal cortex (VMPFC) was activated (in addition to the left posterior superior temporal sulcus, LpSTS), whereas in judgments about the others-tagged stimuli, brain activation was observed primarily in the dorsolateral prefrontal cortex (DLPFC). Furthermore, brain activation in the DLPFC was inversely correlated with the activation in the VMPFC, but it was positively correlated with the activity in the LpSTS. Further source of evidence about the VMPFC involvement on the SPE was found in a study with patients with lesions in the left VMPFC. These patients showed a lower SPE, compared to a control-matched group (Sui et al., 2015). Overall, meta-analyses, neuroimaging, and patient studies suggest that self-relevant stimuli are processed in the VMPFC, while stimuli related to the others have been associated with the DLPFC. Humphreys and Sui (2016) proposed an integrative theoretical framework, the self-attention network, in which three components can be clearly differentiated: a self-representation core linked to the VMPFC; a top-down control component associated with the DLPFC; and finally, a bottom-up orienting component that correlated with the first one, which would be linked to the pSTS.

Up to now, evidence that supports the VMPFC involvement in the SPE is mainly based on correlational neuroimaging studies and just one lesion study. An important limitation of the aforementioned fMRI studies is that they do not allow us to establish a causal link between the activation of these brain areas and the observed behavioral SPE. Thus, whether VMPFC and DLPFC play a causal role in the processing of the self/others, respectively, is still an open question. In this study, we manipulated the neuronal activity by using high-definition transcranial direct current stimulation (HD-tDCS), a neuromodulatory non-invasive brain stimulation (NIBS) technique widely used to assess the causal involvement of specific brain regions in cognitive processes (e.g., Filmer et al., 2013; Bender et al., 2017), including social cognition, and self-related processing (Sellaro et al., 2016; Martin et al., 2017, 2019; Gallo et al., 2018). Based on the self-attention network framework, we hypothesized that, by stimulating the VMPFC through HD-tDCS, self-processing will be affected, compared to the control stimulation condition (sham). Similarly, we also expected that stimulating DLPFC will affect the processing of others, as compared to the sham group. To our knowledge, to date there is only one study that has attempted to partially test these causal hypotheses. In a pre–post design study, Schäfer and Frings (2019) applied 0.5-mA anodal/cathodal tDCS for 20 min over the VMPFC (FPz with reference in F3). They found no modulation in the SPE due to stimulation. However, there are a number of reasons why this result should be interpreted with caution. First, Schäfer and Frings (2019) employed a bipolar montage with electrodes of 9 and 35 cm^2^. This could result in non-focal stimulation and, perhaps, in the undesirable extend of stimulation to other nearby brain areas such as the DLPFC, which has been proven to be crucial for understanding the SPE. Second, they did not include a sham-controlled group and, consequently, we lack the appropriate comparison condition to evaluate the effect of stimulation. To overcome these methodological drawbacks, the present study included a sham condition and employed high-definition montages, which allows more focal stimulation (see Datta et al., 2009; Edwards et al., 2013). To further foster accuracy on focal stimulation, the parameters of these montages were optimized through computational head models (Ruffini et al., 2014). Moreover, we extended the study of Schäfer and Frings (2019) by including the DLPFC as a target for stimulation and also by using online, rather than offline, stimulation. To summarize, we studied whether anodal and cathodal stimulation of the VMPFC or the DLPFC modulates the SPE by using a randomized, double-blind, sham-controlled, parallel group, online stimulation, and multichannel HD-tDCS design.

## Materials and Methods

### Participants

We used G^∗^Power (Faul et al., 2007) to estimate the appropriate sample size to detect a medium effect size *f* = 0.2 (Cohen, 1988), at an alpha level of 0.05 and power (1–β) = 0.95. Although the estimated sample was 70, we increased the sample size to 90 (18 participants per group) to allow counterbalance of both geometric shapes and labels (3 × 3) within each group.

Ninety participants (69 women) ranging in age between 18 and 26 years (*M* age = 20.1 years, *SD* = 2.1) took part in the study. They were naïve to the purpose of the experiment and received course credit for their participation. All had normal or correct-to-normal vision and no history of neurological or psychiatric disorders. They were also screened for the HD-tDCS exclusion criteria (e.g., pregnancy, epilepsy, medication, and use of pacemakers). The study was approved by the University of Murcia ethics committee and conformed to the declaration of Helsinki for human research. Written informed consent was obtained from all participants.

### Procedure

The experiment consisted of a single session of HD-tDCS stimulation while participants performed a task designed to evaluate the SPE. Eighteen participants were randomly assigned to one of the five stimulation conditions (cathodal-VMPFC, anodal-VMPFC, cathodal-DLPFC, anodal-DLPFC, and sham). The task was equivalent to that used by Sui et al. (2012, Experiment 1) and consisted of two stages. In the first stage, participants were asked to remember the associations established between three geometric shapes (triangle, circle, and square) and three labels corresponding to the self, a friend, or a stranger. The shape-label associations were counterbalanced across participants and equally represented in each stimulation condition. In the second stage, participants performed a matching task in which they had to judge whether the different shape-label associations matched or mismatched the previously established associations. Each trial started with the presentation of a 500-ms central fixation cross. Then, a shape and a label were simultaneously presented above and below the fixation cross for 100 ms, followed by a blank screen for 1100 ms. Participants had to indicate, as quickly and accurately as possible, whether the shape and label matched or not by pressing one of two response buttons. Then, a feedback message (“correct” or “incorrect”) appeared during 500 ms and a new trial began. Each participant completed five blocks of 48 trials, for a total of 240 trials. Each block was composed of 8 trials of each shape-label combination (self-matched, self-non-matched, friend-matched, friend-non-matched, stranger-matched, and stranger-non-matched) presented in a random order. Participants completed a practice block of 48 trials with the same distribution as the experimental ones. The stimuli were presented on a 22-in. TFT monitor (resolution = 1920 × 1080 pixels). With a view distance of about 60 cm, the three geometrical shapes were presented subtending 4 × 4 approximately, above the fixation cross (1 × 1). The Spanish words *TÚ* (you), *AMIGO* (friend), and *EXTRAÑO* (stranger) were displayed below the fixation cross, subtending visual angles of about 1.7 high × 1.4, 4, or 4.2 width. The distances between the fixation cross and the center of the shape and the label were 4 and 3, respectively. The background color of the screen was gray, and stimuli were presented in white. Participants responded using a Chronos^®^ response box. The experiment was run using E-Prime 3.0 software (Schneider et al., 2002).

### HD-tDCS

**FIGURE 1:**
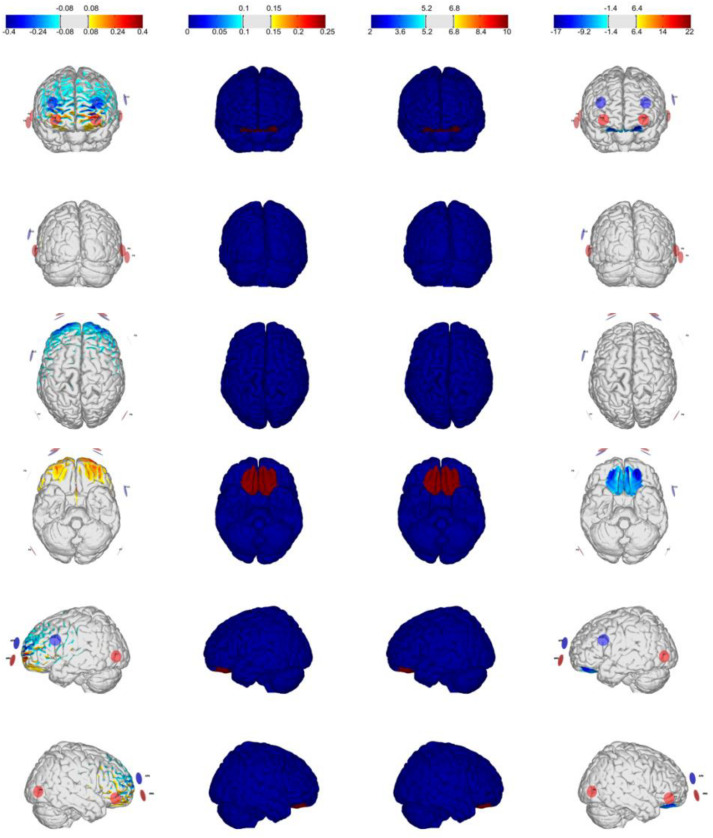
Optimization for Montage 1: VMPFC-anodal (excitation), IMax = 2.0 mA, 8-channel montage. From **left to right:** normal component of the E-field En (V/m), target E-field (V/m), target weight, and the Error Relative to No Intervention (ERNI; mV2/m2) for gray matter (see Ruffini et al., 2014).

**FIGURE 2:**
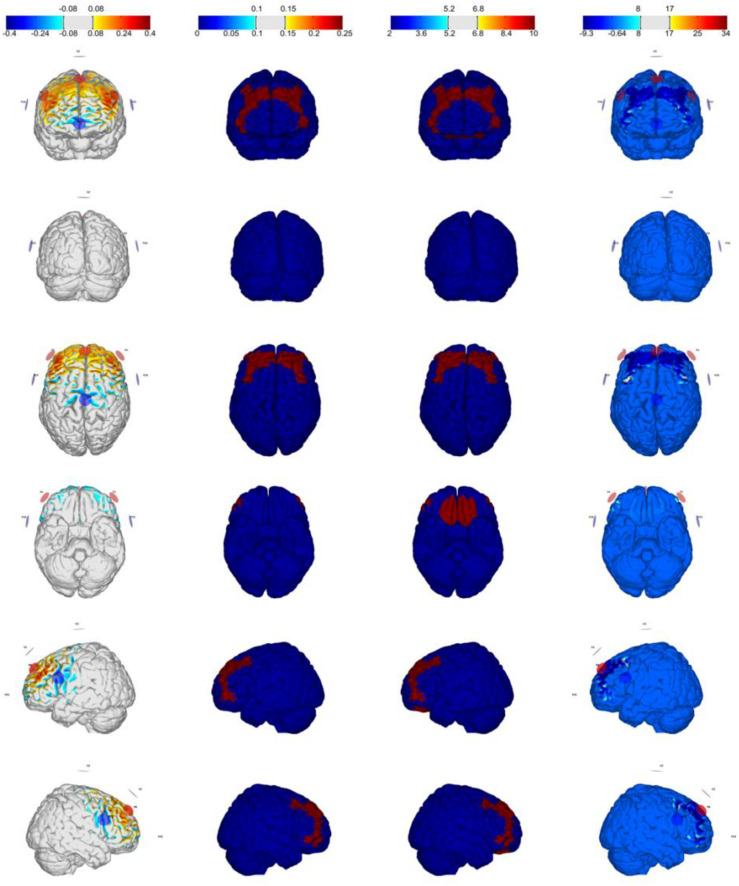
Optimization for Montage 2: DLPFC-anodal (excitation) with maximum weight, IMax = 2.0 mA, 7-channel montage. From **left to right:** normal component of the E-field En (V/m), target E-field (V/m), target weight, and the ERNI (mV2/m2) for gray matter (see Ruffini et al., 2014).

The stimulation was administered using a StarStim^®^ wireless neurostimulator system (Neuroelectrics, Barcelona, Spain). We used four different multichannel stimulation montages for bilateral excitation/inhibition of the VMPFC/DLPFC, optimized using StimWeaver (see Figures 1, 2), a software to model electric fields in the brain generated by tDCS stimulation (Miranda et al., 2013; Ruffini et al., 2014). Table 1 summarizes the electrodes (π cm^2^ Ag/AgCl) positions for VMPFC/DLPFC montages based on the 10–20 international EEG system. The currents (μA) per electrode are for excitatory effects. For inhibitory effects (montage 3: cathodal-VMPFC; montage 4: cathodal-DLPFC), current signs were changed. The maximum current at any electrode was 2.00 mA, and the maximum total injected current was 4.00 mA, with an average electric field (Avg *E*_*n*_) magnitudes of.046 and.071 V/m for excitatory effects in the VMPFC and DLPFC as target areas, respectively. Once the label-shape association learning is finished, stimulation was administered online from the beginning of the matching task (practice block) until the experiment ended (11 min and 30 s approximately), with 30 s ramped up at the start of the stimulation. The sham condition consisted in applying the current of montage 1 only at the ramp period to emulate the skin tingling sensation. Both the researcher and the participants were blinded to the experimental conditions.

**TABLE 1:** Electrode positions for anodal HD-tDCS over VMPFC/DLPFC montages based on the 10–20 international EEG system.

Montage 1: anodal-VMPFC (8-channel)	Montage 2: anodal-DLPFC (7-channel)
AF3: −2,000 μA	F3: 1,528 μA
AF4: −1,798 μA	F4: 1,702 μA
F8: 331 μA	FC5: −977 μA
FC5: −202 μA	FC6: −867 μA
FP1: 1,903 μA	FPZ: −1,145 μA
FP2: 1,160 μA	FZ: 769 μA
P7: 363 μA	Cz: −1,010 μA
P8: 243 μA	

*The currents shown per electrode are for excitatory effects.*

## Results

**FIGURE 3:**
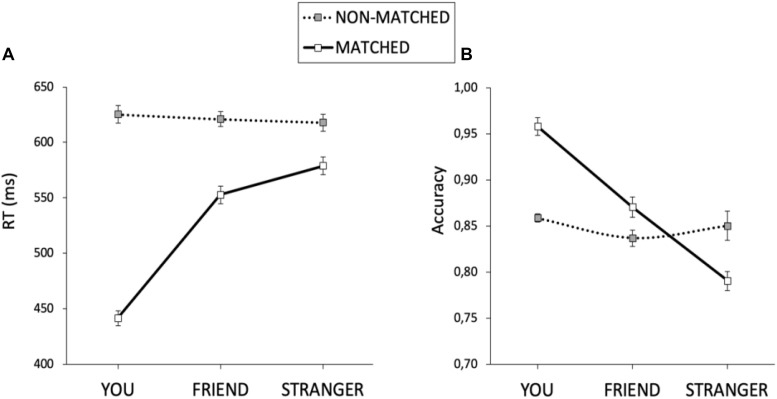
The self association effect for matched and non-matched pairs for RTs **(A)** and accuracy **(B)**. The error bars represent the standard error of the mean.

We excluded trials with no response (2.23%) from both RT and accuracy analyses. Moreover, we excluded from RT analysis trials with incorrect responses (12.51%) and trials with RTs higher and lower than 2.5 semi-interquartile ranges to the median of each participant in each condition (1.51%). Mean RT and accuracy are displayed in Table 2. Mean RTs were submitted to a 2 × 3 × 5 mixed ANOVA with match (matching and non-matching) and shape association (YOU, FRIEND, and STRANGER corresponding to the presented label) as the within-participant factors, and stimulation (cathodal-VMPFC, anodal-VMPFC, cathodal-DLPFC, anodal-DLPFC, and sham) as the between-participant factor. There was a main effect of shape association, *F*(2, 170) = 115.505, *p* < 0.001, and η^2^ = 0.561. Responses for the self association were faster than those for the other two associations (*p*s < 0.001), while RTs for the friend association were faster than those for the stranger association (*p* = 0.045). There was also a main effect of match, *F*(1, 85) = 684.691, *p* < 0.001, and η^2^ = 0.886, revealing shorter RTs on matching than on non-matching trials. The main effect of stimulation was not statistically significant, *F*(4, 85) < 1. The shape association × match interaction reached statistical significance, *F*(2, 170) = 175.704, *p* < 0.001, and η^2^ = 0.661. When matching and non-matching conditions were analyzed separately, we found no effect of shape association for non-matching pairs, *F*(2, 170) = 1.241, *p* = 0.292, and η^2^ = 0.013, whereas there was an effect of shape association in the matching condition, *F*(2, 170) = 200.148, *p* < 0.001, and η^2^ = 0.696 (Figure 3A). Neither the stimulation × shape association nor the stimulation × matching interactions reached statistical significance, *F*(8, 170) = 1.259, *p* = 0.268, η^2^ = 0.009; and *F*(4, 85) < 1, respectively. Finally, and of special interest for the purposes of this work, we found that the shape association × match × stimulation interaction, which would test our hypothesis that VMPFC/DLPFC stimulation modulates the magnitude of self/stranger processing, did not reach statistical significance, *F*(8, 170) = 1.289, *p* = 0.252, and η^2^ = 0.019. Because this lack of interaction is the key result for the present study and following the recommendation of de Graaf and Sack (2018), we used a Bayesian approach to quantify the evidence supporting this interaction. Specifically, we conducted a Bayes factor analysis using JASP 0.11.1 (JASP Team, 2019) with default parameters (Rouder et al., 2012; Morey and Rouder, 2015). Results showed that the observed data were 10.7 times more likely under the model that excluded the shape × match × stimulation interaction (BF_10_ = 4.145 × 10^120^) than under the full model (BF_10_ = 3.867 × 10^119^).

**TABLE 2:** Mean RT and accuracy as a function of shape association, match and stimulation.

Association	Match	Stimulation	RT (Mean)	RT (SD)	Acc (mean)	Acc (SD)
You	Matched	DLPFC-A	429	57	0.86	0.09
		DLPFC-C	454	40	0.81	0.10
		Sham	424	63	0.86	0.09
		VMPFC-A	459	78	0.89	0.08
		VMPFC-C	438	62	0.87	0.10
	Non-matched	DLPFC-A	546	79	0.95	0.05
		DLPFC-C	556	61	0.97	0.4
		Sham	533	92	0.96	0.04
		VMPFC-A	554	71	0.96	0.02
		VMPFC-C	573	73	0.94	0.06
Friend	Matched	DLPFC-A	574	69	0.86	0.12
		DLPFC-C	574	67	0.76	0.11
		Sham	561	95	0.84	0.09
		VMPFC-A	592	64	0.87	0.07
		VMPFC-C	590	78	0.86	0.09
	Non-matched	DLPFC-A	608	65	0.87	0.07
		DLPFC-C	627	56	0.84	0.10
		Sham	605	95	0.88	0.08
		VMPFC-A	640	70	0.88	0.09
		VMPFC-C	645	70	0.87	0.08
Stranger	Matched	DLPFC-A	619	58	0.82	0.12
		DLPFC-C	611	53	0.82	0.12
		Sham	608	87	0.87	0.08
		VMPFC-A	642	67	0.88	0.08
		VMPFC-C	624	58	0.70	0.07
	Non-matched	DLPFC-A	598	55	0.79	0.18
		DLPFC-C	611	68	0.78	0.15
		Sham	622	91	0.78	0.14
		VMPFC-A	631	73	0.79	0.12
		VMPFC-C	626	62	0.82	0.16

*RT = reaction time; Acc = accuracy; and SD = standard deviation.*

**FIGURE 4:**
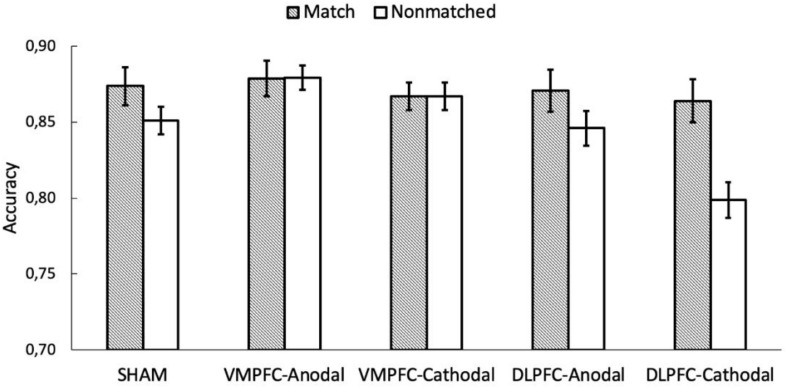
The y axis represents the accuracy on shape-label matching task as a function of matching and the stimulation conditions. The error bars represent the standard error of the mean.

An equivalent ANOVA on the percentages of correct responses revealed a main effect of shape association, *F*(2, 170) = 59.165, *p* < 0.001, and η^2^ = 0.397. Responses for the self association were more accurate than those for the other two associations (*p*s < 0.001), and responses for the friend association were more accurate than those for the stranger association (*p* < 0.001). There was also a main effect of match, *F*(1, 85) = 20.209, *p* < 0.001, and η^2^ = 0.166, revealing higher accuracy on matching trials than on non-matching trials. The main effect of stimulation was not statistically significant, *F*(4, 85) = 1.369, *p* = 0.251, and η^2^ = 0.008. Importantly, there was a significant interaction between shape association and match, *F*(2,170) = 35.315, *p* < 0.001, and η^2^ = 0.289. Separated analyses for the matching and non-matching conditions showed no effect of shape association for the non-matching condition, *F*(2, 170) = 1.863, *p* = 0.158, and η^2^ = 0.020, but there was an effect of shape association for matching trials, *F*(2, 170) = 76.409, *p* < 0.001, and η^2^ = 0.466 (Figure 3B). The stimulation × shape association interaction did not reach statistical significance, *F*(8,170) < 1. Importantly, as with RTs, the shape association × match × stimulation interaction failed to reach statistical significance, *F* < 1. Congruently, Bayes factor analysis showed that the observed data were 35.9 times more likely under the model that excluded the shape × match × stimulation interaction (BF_10_ = 3.344 × 10^29^) than under the full model (BF_10_ = 9.306 × 10^27^). So far, all these results paralleled those previously described for RTs. However, accuracy analysis revealed an unexpected match × stimulation interaction, *F*(4, 85) = 4.213, *p* = 0.004, and η^2^ = 0.138. Separated analyses for the matching and non-matching conditions revealed a main effect of stimulation for non-matching trials, *F*(4, 85) = 3.441, *p* = 0.012, and η^2^ = 0.139, but not for matching trials, *F* < 1. An inspection of Figure 4 suggests that the effect of stimulation on non-matching trials resulted from lower accuracy with cathodal-DLPFC stimulation. Congruently with this observation, the effect of stimulation in the non-matching condition vanished when the cathodal-DLPFC group was removed from the analysis, *F* < 1.

## Discussion

In the present study, we evaluated the SPE through an unbiased task in which participants first associated three different arbitrary shapes with labels for themselves, a friend, or a stranger and then judged whether subsequent label-shape pairings matched these previously learned associations. We found a large SPE for both RT and accuracy data. These results replicate previous findings of self-bias in the perceptual domain using the shape-label matching paradigm (e.g., Sui et al., 2012, 2013; Sun et al., 2016; Sui and Gu, 2017; Nijhof et al., 2020). At the neural level, previous neuroimaging findings with the shape-label matching task involved two crucial brain areas in the so-called self-attention network (Humphreys and Sui, 2016). The VMPFC was involved in the cortical representation of the self, whereas the DLPFC was involved in the top-down control required to represent others (Denny et al., 2012; Wagner et al., 2012; Sui et al., 2013).

In searching for a causal role of areas in the self-attention network on the modulation of the SPE, Schäfer and Frings (2019) stimulated the VMPFC using a pre–post design, anodal vs. cathodal tDCS stimulation of 0.5 mA during 20 min, over FPz and F3, and found no evidence of any modulation. Here, we aimed to assess causal effects on self-/other-relevant stimulus processing in a perceptual matching task not only of the VMPFC but also of the DLPFC, as both areas are crucial components of the self-attention network. However, contrary to Schäfer and Frings (2019), we implemented an advanced methodological HD-tDCS setup with four different multichannel montages. To avoid overlaps in DLPFC/VMPFC tDCS stimulation, we performed a simulation based on computational modeling of electric current densities implemented on realistic head model (Ruffini et al., 2014).

Based on Sui et al. (2013), we hypothesized that tDCS stimulation over the VMPFC would bring about alterations in the processing of the self-related stimuli, while tDCS stimulation over the DLPFC would cause alterations in the processing of the stranger-related stimuli. The results showed that stimulation did not succeed to modulate the magnitude of the SPE, as evidenced by the non-significant interaction involving the three factors, neither for RT nor for accuracy data. Because the lack of a three-way interaction is a key result for the present study, we used a Bayesian approach (Bayes factor) for interpreting null results in a more robust and quantitative statistical fashion, as it is common in NIBS studies (Biel and Friedrich, 2018; de Graaf and Sack, 2018). The Bayesian analysis showed strong evidence for null effects of a single-session of HD-tDCS stimulation over VMPFC/DLPFC in selectively modulating the SPE. Our null results are consistent with those of Schäfer and Frings (2019) and extend the lack of causal effects to the DLPFC, despite that the brain area had been identified in previous fMRI studies and in cognitive models as a key hub for understanding the SPE (Sui et al., 2013; Humphreys and Sui, 2016).

Despite the difficulty in interpreting null results, it is likely that the aforementioned associations between VMPFC/DLPFC and self–other processing reported in fMRI bold signals constitute a sort of epiphenomenon, that is, the consequence of cross-activation between those brain areas and others that may actually underlie the SPE. For instance, there is evidence that the LpSTS plays a role in detecting the social salience of external stimuli that help infer the mental state of others (Allison et al., 2000) and is associated with perceptual matching of self-stimuli in a coupling with the VMPFC (Sui et al., 2013). In a similar task to the one used here, Sui et al. (2013) found that the stronger the effective connection from VMPFC to LpSTS, the faster and more accurate the responses observed for self-shape matching trials. The relevance of LpSTS in causing the SPE is further demonstrated by the fact that this area is activated by both the self-related shape and the self-related label, whereas the VMPFC is activated only by the self-related label. These results suggest that activation of the LpSTS along with the VMPFC is crucial to observing causal effects on the SPE. A second candidate is the temporo-parietal junction (rTPJ), as this area plays a crucial role in dissociating self- and other-related processes (Santiesteban et al., 2012; Martin et al., 2019), as well as in inhibiting the processing of the self to facilitate the processing of others (Soutschek et al., 2016; Payne and Tsakiris, 2017). Further studies should focus on assessing whether the LpSTS and the rTPJ are the actual cause of the SPE in perceptual matching tasks.

Although we found that tDCS over VMPFC/DLPFC failed to selectively modulate the processing of self-/other-related stimuli, further analyses of the match × stimulation interaction showed that only cathodal stimulation over the DLPFC, which is supposed to inhibit cortical activity, led to a drop-off in accuracy in non-matching trials. Note that some neuroimaging studies have associated the DLPFC with performance in conflict tasks (e.g., Stroop and flanker tasks), mainly when incongruent trials are presented (Cieslik et al., 2015; Song et al., 2017). In the same line, cathodal tDCS over the DLPFC has been shown to modulate performance on tasks involving cognitive (Frings et al., 2018; Baumert et al., 2020) as well as emotional (Martínez-Pérez et al., 2019) conflict, which reinforces the assumption that the frontal area plays a causal role in conflict resolution. Importantly, the non-matching condition here might share some commonalities with the incongruent condition in conflict tasks. As with incongruent trials, there is a cost in RTs and correct responses to stimuli in non-matching compared to matching trials, likely due to a conflict between the learned shape-label associations and the incongruent ones displayed in non-matching trials. Although costs in non-matching trials were observed with both RTs and percentage of correct responses, cathodal inhibition of the DLPFC affected correct responses only. These results extend the role of the DLPFC to conflict-like tasks that do not involve any kind of response-based competition but instead require the participant to react to shape-label combinations that contradict the ones previously learned.

Finally, we cannot rule out that a different design for electrical brain stimulation that controls for individual differences in brain and cognition (e.g., see Kanai and Rees, 2011; Seghier and Price, 2018) or uses transcranial alternating, instead of direct, current stimulation, tACS (see Harty and Cohen-Kadosh, 2019) is more likely to find causal relationships between DLPFC/VMPFC and the SPE. On this scenario, the present study is a further piece on the exploratory path to the standardization of protocols to achieve more reliable effects of electrical brain stimulation on cognition, and therefore, it may contribute to clarifying this puzzling pursuit by suggesting follow-up studies. We have shredded insights about ineffective combinations of certain tDCS parameters for modulating the SPE, while overcoming sensible weakness of previous studies. Moreover, given the issues of reproducibility in cognitive neurosciences (Szucs and Ioannidis, 2017), and the current state of the art of NIBS, with non-trivial evidence collected that challenges reliable effects of a single session of tDCS on behavior (Bestmann et al., 2015; Horvath et al., 2015; Parkin et al., 2015; Medina and Cason, 2017; Galli et al., 2019), the call for dissemination of results like the ones reported here becomes paramount (Filmer et al., 2019).

## Data Availability Statement

The raw data supporting the conclusions of this article will be made available by the authors, without undue reservation.

## Ethics Statement

The studies involving human participants were reviewed and approved by University of Murcia ethics committee. The patients/participants provided their written informed consent to participate in this study.

## Author Contributions

VM-P: conceptualization, methodology, software, formal analysis, investigation, and writing – original draft preparation. GC: conceptualization, methodology, formal analysis, and writing – original draft preparation, reviewing and editing. LP: investigation and writing – original draft preparation. LF: conceptualization, methodology, writing – original draft preparation, reviewing and editing, and funding acquisition. All authors contributed to the article and approved the submitted version.

## Conflict of Interest

The authors declare that the research was conducted in the absence of any commercial or financial relationships that could be construed as a potential conflict of interest.
